# Predictors of postoperative complications after sternectomy on oncologic patients

**DOI:** 10.1016/j.clinsp.2024.100468

**Published:** 2024-10-16

**Authors:** João Paulo Cassiano de Macedo, Pedro Henrique Xavier Nabuco-de-Araujo, Benoit Jacques Bibas, José Ribas M. de Campos, Paulo M. Pêgo-Fernandes, Ricardo M. Terra

**Affiliations:** a Thoracic Surgery Department, Instituto do Coração (InCor), Hospital das Clínicas da Faculdade de Medicina da Universidade de São Paulo (HCFMUSP), São Paulo, SP, Brazil; b Thoracic Surgery Department, Instituto do Câncer do Estado de São Paulo (ICESP), Hospital das Clínicas da Faculdade de Medicina da Universidade de São Paulo (HCFMUSP), São Paulo, SP, Brazil

**Keywords:** Chest wall tumor, Chest wall resection, Reconstruction

## Abstract

*Background:* Chest wall tumors are uncommon. The surgical objective is local disease control and the relief of symptoms. Due to the heterogeneity of cases, the great variety of reconstructions, size of resection, and clinical and surgical outcomes are still uncertain.

*Methods:* Patients were submitted to sternectomies for tumors between 1997 and 2019. Oncological, and surgical characteristics were taken into consideration. The outcomes were related to the size of resection and classified into local and systemic complications. The authors used univariate and multivariate analyses to determine predictors of complications. Survival analysis and Kaplan-Meier curves were obtained.

*Results:* Thirty resections were performed due to metastatic disease. Partial sternectomy was accomplished in 48.8 %, followed by subtotal in 40 %. Primary closure using Pectoralis major was performed in 48.8 %, and Latissimus dorsi in 35.5 %. Polypropylene mesh was used in 86.6 % of reconstructions. The prosthesis removal was necessary in 6.66 %. Respiratory failure was evidenced in 6.66 %. The resection area was a predictor of local and systemic complications (p = 0.0029; p = 0.0004 respectively) in univariate analysis. However, the size of resection was the only predictor of systemic complications regarding multivariate analysis (p = 0.014, 95 % CI 1.00‒1.07).

*Conclusion:* The size of the resection was related to systemic complications, and the mesh reconstruction resulted in a low percentage of prosthesis removal and respiratory failure. This suggests a high resistance to local issues and a low respiratory failure rate.

## Highlights

• Chest wall tumors are rare conditions.
• The literature has poorly documented the Chest wall reconstruction outcomes based on polypropylene mesh.
• The postoperative evaluation resulted in a few cases of prostheses removal and a low respiration failure rate.

## Introduction

**Fig. 1:**
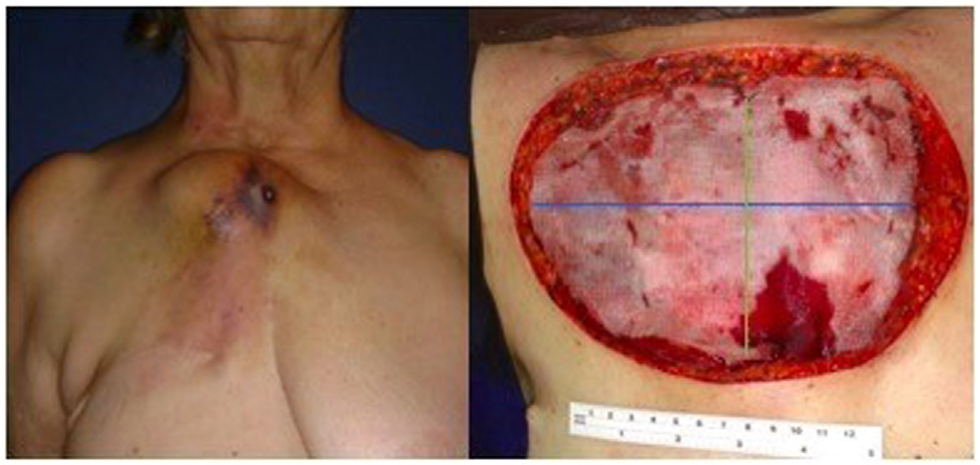
(A) Metastatic chest wall tumor after mastectomy. (B) Defect size after resection. The blue line represents the side-to-side measure, and the green line represents the top-to-bottom measure.

Primary chest wall tumors represent 1 %‒2 %^1^ of all thoracic neoplasms. On the other hand, metastatic lesions are much more frequent. For instance, the local disease relapse after mastectomy or breast-sparing surgery can vary from 5 % to 40 % (Fig. 1).^2^

Patients frequently complain about pain and a palpable mass. The objective of Full-Thickness Chest Wall Resection (FTCWR) is to provide local control of primary or recurrent disease (oncological purpose) and long-term palliation. The non-curative intent should be taken into consideration in cases of intractable pain, infection, or ulcerated skin.

As the main surgical goal may vary, the preoperative characteristics can be diverse due to the heterogeneity of the population. In addition, the association between these aspects and the type of reconstruction, size of resection, and clinical and surgical outcomes, is still uncertain. Therefore, the objective of this study is to evaluate the predictors of postoperative outcomes in patients who have undergone oncological sternectomy in the Brazilian population.

## Material and methods

The authors retrospectively assessed the institutional chest wall database from 1997 to 2019. The study took place at an oncology reference center hospital in the Brazilian public health system.

The inclusion criteria were subjects who underwent oncologic sternectomy due to palliative or curative intent. The authors excluded sternal resections in response to infectious diseases, and sternotomy wound complications after non-cancer surgery. All patients underwent a CT scan for diagnosis and surgical planning. An MRI was necessary in cases of uncertain CT findings. A PET-Scan was used to evaluate systemic/metastatic disease, when necessary. A pre-operative core biopsy was performed in the majority of patients. Most cases were discussed by a multidisciplinary board.

Surgical margins were taken 4‒5 cm in cases of curative intent, and 2 cm or macroscopically disease-free in cases of non-curative resections. The authors classified sternectomy according to Fabre et al.^3^ as total (combining manubrium, body and xiphoid); subtotal (when > 90 % of the longitudinal diameter was resected), and partial (when < 90 % of the longitudinal measure was resected).

The authors classified the type of prosthesis into: none, Polypropylene Mesh (PPM), bovine pericardium, and titanium bars. The myocutaneous flaps applied in reconstruction were pectoralis major, latissimus dorsi, abdominal, and thigh flap. The resection area was intraoperatively obtained and considered as a square shape. The size was calculated as the largest side-to-side measure, multiplied by the top-to-bottom measure (Fig. 1). Local and systemic complications were categorized according to the Common Terminology Criteria for Adverse Events version 5.0.^4^

Mortality was considered during the hospital stay and the follow-up. Overall survival was defined as the time from sternal resection to death. Disease-free survival was characterized as the time from sternal resection to either local or distant recurrence.

STATA 14 software (Stata Corporation, College Station, TX, USA) was used for all statistical analyses. Normal distribution was assessed, and Fisher's exact test was used for categorical data. Mann-Whitney was used as a univariate analysis and logistic regression for multivariate analysis. The authors applied the Spearman coefficient for the correlation between variables. The authors performed the Kaplan-Meier curve and the Cox regression model for the survival analysis. The alpha error was set at 5 %.

## Results

A total of 45 patients were included in this study. Most of the patients were female, 44 %. Demographic data is summarized in Table 1. Metastatic tumors accounted for the majority of the cases (n = 66, 67 %), with breast cancer being the most common etiology. (Table 2). The authors performed partial sternectomy in 48.8 % of the cases, followed by subtotal in 48 %. Pectoralis major was the most frequent myocutaneous flap, being used in 48.8 %, of cases. PPM was used in 86.6 % (Table 2). Preoperative skin ulceration appeared in 28.8 % of patients and it was not considered a contraindication for mesh use. Thirteen subjects had already undergone previous operations, ten of whom as breast surgery. The mean time between the first procedure and FTCWR was 16.73 months (SD ± 38.14). FTCWR was redone in two cases.

**Table 1:** Demographic DATA and comorbidities.

Characteristic	Number (%)
Total	45 (100)
Female	29 (64.44)
Male	16 (35.55)
Age (mean, SD)	55.51 ± 13.27
KPS (median, range)	100 (60‒100)
ECOG (median, range)	ECOG: 0 (0‒2)
Smoking	17 (37.78)
Hypertension	10 (22.22)
Alcoholism	3 (6.67)
Diabetes	3 (6.67)
COPD	2 (4.44)
Asthma	2 (4.44)
Hepatitis	2 (4.44)
Dyslipidemia	2 (4.44)
Obesity	2 (4.44)
CAD	1 (2.22)
Stroke	1 (2.22)
Dementia	1 (2.22)
HIV	1 (2.22)
Parkinson	1 (2.22)
CKD	1 (2.22)
Hypothyroidism	1 (2.22)
Cerebrovascular Disease	1 (2.22)
Heart Stroke	1 (2.22)

COPD, Chronic Obstructive Pulmonary Disease; CAD, Chronic Arterial Disease; HIV, Human Immunodeficiency Virus; CKD, Chronic Kidney Disease.

**Table 2:** Summary of complications and surgical DATA.

Local complications	Number (%)
Necrosis (partial or total)	5 (11.1)
Seroma (operative wound or donor area)	3 (6.6)
Infection + mesh removal	3 (6.6)
Infection	2 (4.4)
Dehiscence	2 (4.4)
**Systemic complications**	**Number (%)**
Pneumothorax	4 (8.8)
Sepsis	2 (4.4)
Delirium	2 (4.4)
Pneumonia	2 (4.4)
Pain	1 (2.2)
Atelectasis	1 (2.2)
Pulmonary embolism	1 (2.2)
Chylothorax	1 (2.2)
Pleural effusion	1 (2.2)
Acute abdomen	1 (2.2)
Tracheostomy	1 (2.2)
Stroke	1 (2.2)
**Sternectomy**	**Number (%)**
Partial	22 (48.88)
Subtotal	18 (40.0)
Total	5 (11.11)
**Prosthesis**	**Number (%)**
PPM	39 (86.66)
Osteosynthesis +PPM	3 (6.66)
None	2 (4.44)
Pericardium	1 (2.22)
**Myocutaneous flap**	**Number (%)**
*Pectoralis major*	22 (48.88)
*Latissimus dorsi*	16 (35.55)
Abdominal	6 (13.33)
Thigh	1 (2.22)

PPM, Polypropylene Mesh.

Local complications occurred in 33.33 %, mainly represented by partial graft necrosis, and seroma (Table 2). Local infection was evidenced in 5 cases, with mesh removal being necessary in three. Systemic complications occurred in 40 %, mainly represented by pneumothorax. The most feared complication regarding FTCWR is respiratory failure. It was noted in three cases, resulting in two deaths. The first patient required mechanical ventilation and reoperation with another steel bar placement, making mechanical ventilation weaning possible. The second case was submitted to an uneventful extended FTCWR. The respiratory failure occurred after local complications and several dressing changes related to negative pressure wound therapy. Finally, the third FTCWR was performed alongside the resection of a large cervicothoracic desmoid tumor. A deep cervical dissection was required, resulting in a laryngeal nerve injury. A tracheostomy was necessary due to difficult weaning ventilation. Deaths during the same hospital stay were only 6 %.

**Fig. 2:**
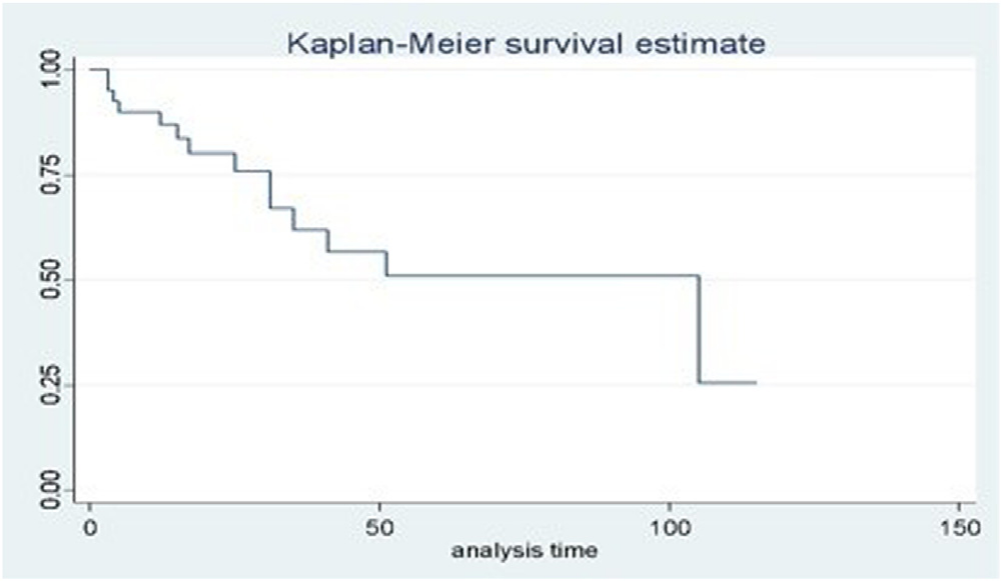
Kaplan-Meier curve.

The size of the chest defect was related to local and systemic complications (p = 0.0029; p = 0.0004 respectively). Nonetheless, the univariate analysis did not show significance relating to the compromised surgical margin with local disease relapse (p = 0.682), and tumor infection with prosthesis removal (p = 0.650). The positive relation between disease-free time and survival time was reinforced by the Spearman coefficient (0.87, p = 0.00). Multivariate analysis, regarding histology, local infection, previous resection or radiotherapy, chemotherapy, myocutaneous flap and synthetic material. showed the resection area as a predictor of systemic complications (p = 0.014, 95 % CI 1.00‒1.07. Statistical significance was not achieved regarding the size of resection as a local complication predictor. The mean period between sternectomy and local recurrence was 4.8 months (SD ± 14.79), and the mean disease-free time was 22.71 months (SD ± 29.60). During the follow-up period of 22 years, the mortality rate was 40 %, probably as a result of metastatic tumors. The mean survival time was 29.22 months (SD ± 29.77). A Cox-regression model was assessed, even though not statistically significant, in addition to the Kaplan-Meier curve (Fig. 2).

## Discussion

Sternectomy is considered a complex procedure, frequently performed in a heterogeneous group of patients. Even though the studied population is quite diverse the resection area is the main predictor of local and systemic complications. Furthermore, regression analysis reaffirms extensive resections as a systemic complication agent but not local. The authors could only suggest its relation to local complications, due to the p = 0.076, probably because of the small sample size. The authors completely agree with Weyant M. J et al.^5^ as the analysis showed the size of the chest wall defect significantly predicted postoperative complications.

Total sternectomy corresponded to only 11.11 %. The decision about the muscle flap must be made jointly by the thoracic and plastic surgical teams, regarding the chest wall defect and location. The latissimus dorsi was not the most used in this series, but it was the workhorse flap in extended chest wall resections. This graft has a good muscle-skin association, in addition to the unnecessity of internal mammary vessel integrity. Petrella F, et al.^2^ indicated their preference for using latissimus dorsi in extended CWR in the treatment of breast cancer recurrence so do we.

Reconstruction plays an important role in FTCWR. Despite the massive use of PPM, statistics did not associate it with 33 % of local complications. These are the most frequent obstacles in FTCWR, despite the variety of prostheses. The ideal prosthetic has already pointed characteristics: sufficient strength, good biocompatibility, flexibility, and radiolucency to allow radiographic follow-up. Titanium plates are good alternatives as they can provide good chest rigidity, despite bar fractures in 11 %^6^ of cases. The association with bone allograft could be feasible, as the literature has already reported good outcomes. The main difficulty is the necessity of a tissue bank and specific legislation. The sandwich technique, composed of PPM and methyl methacrylate, is considered a rigid reconstruction. It was first published by McCormack P et al.,^7^ and modified by Weyant M. J et al.^5^ Compared to PPM, and PTFE, the sandwich did not show a significant difference in respiratory complications. On the other hand, it was associated with a higher number of wound complications.

The mortality rate was 40 % during the 22-years. Butterworth J. A, et al.^8^ reported a 49 % oncologic mortality rate in 18 months, with a predominance of metastatic tumors (63 %). According to Ahmad U, et al.^9^ The recurrence-free Probability (RFP) was similar between breast cancer patients who received R0 resection and those who received R1/2. The authors also could not relate *non*-R0 resections to local disease relapse, probably because of the small sample. Despite the great majority of metastatic cases, the mean survival and disease-free times were 29.22 and 22.71 months respectively. Bongiolatti S, et al.^10^ published the overall survival at 5 and 10 years was 59 % and 40 %, respectively, and disease-free survival at 5-years was 61 % principally represented by primary sternal tumors.

Given the limitations of this study, the retrospective data, and single-center experience, a multicenter study based on large datasets is still necessary.

This study affirms the defect size as a predictive factor for systemic complications. It also suggests that PPM is an effective and safe choice for extensive FTCWR. The authors used PPM in 86.6 % of the cases, matched with 28.89 % of preoperative tumor infections. The prosthesis removal was necessary in 6.66 % of patients. Herein the authors highlight the resistance to local issues, the absence of a relation between systemic complications, and the low respiratory failure rate.

## Conflicts of interest

The authors declare no conflicts of interest.

## Appendix Supplementary materials

Image, application 1
