# Supplementary material for: Predictors of postoperative complications after sternectomy on oncologic patients
Source: Clinics (Sao Paulo). 2024 Oct 16;79:100468. doi: 10.1016/j.clinsp.2024.100468 (PMC11530808; doi:10.1016/j.clinsp.2024.100468)
Supplement: Supplementary file 1 [file mmc1.docx]

CLINICS-D-24-00009_Supplementary Material

**Supplementary** **Table** Histologic classification.

| **Histologic classification** | **Number (%)** |
| --- | --- |
| ***Primary tumors*** | ***15 (33.33)*** |
| Sarcoma | 7 (15.55) |
| Plasmocytoma | 2 (4.44) |
| Squamous cell | 1 (2.22) |
| Basal cell carcinoma | 1 (2.22) |
| Granular cell tumor | 1 (2.22) |
| Desmoid | 1 (2.22) |
| Bone tumor (non-sarcoma) | 1 (2.22) |
| Neuroendocrine differentiated tumor | 1 (2.22) |
| ***Metastatic*** | ***30 (66.66)*** |
| Breast cancer | 21 (46.66) |
| Triple-negative breast cancer | 3 (6.67) |
| Non-breast metastatic tumors | 9 (20) |
| Lung | 3 (6.66) |
| Germ cell | 1 (2.22) |
| Limb sarcoma | 1 (2.22) |
| Kidney | 1 (2.22) |
| Head and neck squamous cell | 1 (2.22) |
| Thyroid | 1 (2.22) |
| Bile duct cancer | 1 (2.22) |

**Supplementary** **Table** Surgical Data.

| **Sternectomy** | **Number (%)** |
| --- | --- |
| Partial | 22 (48.88) |
| Subtotal | 18 (40.0) |
| Total | 5 (11.11) |
| **Prosthesis** | **Number (%)** |
| PPM | 39 (86.66) |
| Osteosynthesis +PPM | 3 (6.66) |
| None | 2 (4.44) |
| Pericardium | 1 (2.22) |
| **Myocutaneous flap** | **Number (%)** |
| Pectoralis major | 22 (48.88) |
| Latissimus dorsi | 16 (35.55) |
| Abdominal | 6 (13.33) |
| Thigh | 1 (2.22) |

PPM, Polypropylene Mesh.

**Supplementary** **Table** Statistical analysis.

| **Variable** | **Systemic complications (outcome)** | **Local complications (outcome)** |
| --- | --- | --- |
| Chest wall defect (univariate analysis) | p = 0.0004 | p = 0.0029 |
| Chest wall defect (multivariate analysis) | p = 0.014 | p = 0.076 |
|  | 95% CI 1.00‒1.07 | 95% CI 0.99‒1.08 |
| Surgical margin | Local disease (p = 0.682) | |
| Tumor infection | Prosthesis removal (p = 0.650) | |

CI, Confidence Interval.
